# POSITIVE: experiences of an intervention aiming for reversing and preventing frailty using a home monitoring and communication platform within primary health care

**DOI:** 10.1186/s12877-024-04990-7

**Published:** 2024-04-30

**Authors:** Linda Timm, Susanne Guidetti, Marina Taloyan

**Affiliations:** 1 Division of Occupational Therapy, Department of Neurobiology Care Sciences and Society, Karolinska Institutet, Alfred Nobels Alle 23, B4, Huddinge, Stockholm 141 83, Stockholm, Sweden; 2 Medical Unit Occupational Therapy and Physiotherapy, Women´s Health and Allied Health Professionals Theme, Karolinska University Hospital, Stockholm, Sweden; 3 Division of Family Medicine and Primary Care, Department of Neurobiology Care Sciences and Society, Academic Primary Healthcare Centre, Karolinska Institutet, Region Stockholm, Sweden

**Keywords:** Aging, Physical activity, Prevention, Technological tools, Older persons, Qualitative, Occupational therapy

## Abstract

**Background:**

Frailty is increasing worldwide as the population ages. Physical activity is one component that has been shown to hinder and even reverse the process. The POSITIVE system (i.e., maintaining and imPrOving the intrinSIc capaciTy Involving primary care and caregiVErs) is a prevention program that consists of home-monitoring equipment and a communication platform to support the initial treatment of frailty symptoms in primary health care. The participants, older aged (+ 70) frail persons and those at risk for frailty, took part in the program that promoted physical activity at home for six months. The aim was to explore and describe older persons’ experiences of participating in a new prevention program using the POSITIVE system including technological tools intended to prevent the development of frailty.

**Methods:**

Nine interviews were conducted about experiences of participating in an intervention including use of technological tools to support physical activity. Qualitative content analysis was applied.

**Results:**

Two themes revealed: (1) Perceptions of being old are seldom positive, and (2) A rewarding and fruitful participation in the project with suggestions for improvement. Becoming older was related to physical pain and tiredness reducing the performance of former meaningful activities as well as an increase in mental stress. There was also a tendency to postpone the start of everyday activities, and in general, fewer activities were planned for one day than at younger ages. Participating in a physical activity intervention, including the use of technical tools, was considered meaningful and added motivation for engaging in other physical activities, this despite some difficulties with technical tools provided by the program. The contact with health care and the research team was appreciated. In addition, contact with other participants was requested and reported to be highly valued if added to the intervention, which could have been an expression of loneliness.

**Conclusion:**

Participation in a prevention program motivated activities and social interaction. Adding opportunities for participants to meet each other is suggested for improving the intervention in terms of increasing the social dimensions. Our findings conclude that despite difficulties with handling the technical tools for the home-monitoring and communication platform, participation in the POSITIVE intervention was in general a positive experience.

**Supplementary Information:**

The online version contains supplementary material available at 10.1186/s12877-024-04990-7.

## Introduction

Along with the aging population worldwide, the prevalence of geriatric syndrome caused by frailty is increasing [1]. Frailty is associated with reduced physical capacity, feelings of exhaustion, low muscle strength, low walking speed, and unintended weight loss [1, 2]. Physical and mental health combined with social networks and financial stability have been identified as key elements for quality of life from the perspective of older persons [3], which indicate a lower quality of life for people with frailty [4, 5]. Although no clear consensus on the concept of frailty has been reached, a systematic review concluded that frailty comprises five components: vulnerability, genesis, characteristics, phenotypes, and adverse health outcomes [4]. The conclusions from the paper are in line with the WHO definition: “a progressive age-related decline in physiological systems that results in decreased reserves of intrinsic capacity, which confers extreme vulnerability to stressors and increases the risk of a range of adverse health outcomes” [6]. Evidence shows that women tend to be frailer than men in age-matched cohorts, despite women having longer life expectancy [7]. In general, older persons report greater satisfaction with the healthcare they receive, but the effects of gender, ethnicity, and socioeconomic status are equivocal [8]. Studies focusing on frail persons’ satisfaction with health care specifically are scarce.

Frailty is reversible [9, 10], and extensive evidence has shown that physical activity interventions are key for preventing frailty among older people [5, 9, 11–13]. It is also evident that the performance of activities of daily living (ADL) for frail people is improved by physical activity [5, 13]. Lifestyle interventions promoting self-management have been found to be effective at improving physical activity levels [14, 15]. Although there is still a knowledge gap around what type of physical activity is preferred in terms of effectiveness for people with frailty [5, 11], interventions for increasing physical activity for older frail persons are suggested for preventing, delaying, and reversing frailty [1, 12].

Home-based exercises including technological support are applicable and feasible interventions for preventing dependency decline in frail older adults [16]. Evidence shows that technology can be used beneficially for increasing exercise in daily life for persons at older ages [17]. However, the ability to manage everyday technologies such as electronic and technical devices (computers, mobile phones, coffee machines etc.) has been reported to decline for older adults [18]. This decline is even more apparent in people with cognitive impairments and dementia, such as Alzheimer’s disease [18, 19]. The same results can be seen in the persons’ perceived difficulties in using technology, which has also been reported to be high among older persons in general [20, 21]. In addition, age-related changes such as vision and hearing loss and fine motor difficulties add to barriers to the use of technological devices [22].

Despite difficulties in using technical devices for older persons, the digitalization of healthcare services is increasing and developing rapidly. In line with the findings of a literature review showing that technology use can decrease social isolation for older adults [23], the digitalization and availability of technical solutions have also been suggested to increase social participation for older adults in the Swedish context [24]. Several studies have been testing using technology for the prevention and treatment of various chronic conditions and have shown the benefits of introducing such innovations [25].

The POSITIVE system (i.e., maintaining and imPrOving the intrinSIc capaciTy Involving primary care and caregiVErs) is a prevention program that includes an intervention and support that consists of home-monitoring equipment with mobile measurement tools and a communication platform connected with primary health care [26]. The intervention was built on exercises from the Vivifrail program [27], allowing for individual adjustments of levels depending on frailty, functional capacity, and risk of falls. The program included balance and strength exercises as well as walking and stretching. There are a total of six program levels [27], and these can be adjusted along with participation in the program. A more detailed description can be found in a published study protocol [26].

This study was nested in a larger research project as European collaboration (EIT project) designed to evaluate the POSITIVE system in Spain, Poland and Sweden. In Sweden the intervention was tested in Hässelby Academic Primary Health Care Centre (APHCC) in Stockholm. The original aim of the POSITIVE was to promote physical exercise in their home environment in older individuals aged 70 + years. The program used home-monitoring equipment including physical tests, questionnaires and the Vivifrail exercises at the participants’ homes that were monitored by a nurse from Hässelby APHCC using a communication platform via tablet. The measurements included a gait speed placed on the floor, a device to measure power in the lower limbs from the chair stand test and a weighing scale. For finding out the future need and suitability of implementing support for physical activity in home environment, a qualitative study of the experiences of the participation in the program was needed.

The aim was to explore and describe older persons’ experiences of participating in a new prevention program using the POSITIVE system including technological tools intended to prevent the development of frailty.

## Methods

### Design

A qualitative inductive approach was used to obtain an understanding of the participants’ experiences taking part in and using technological tools for performing physical activity within the POSITIVE intervention system at home. Data collection was conducted through individual semi-structured interviews with open-ended questions and analyzed by conducting qualitative content analysis [28, 29].

### Participants and study context

Participants from the Swedish arm of the project POSITIVE were recruited by purposive sampling, where all persons who had finalized the prevention program using the POSITIVE system for six months were asked to participate in an individual interview (*n* = 11). The inclusion criteria for the study were older adults at risk of frailty, as measured by validated screening tools such as the Linda Frieds’ criteria and the Frailty Trait Scale (FTS-5) [30, 31]. One person was excluded from this part of the study due to limited language skills in Swedish where a need for an interpreter would have been necessary, and one participant declined to participate due to limited physical and mental strength. In total, nine participants were included in the study sample (see table 1). Most of the participants included in the study were native-speaking Swedes aged 74 to 85 years. The majority (7/9) of the participants were male. The system was implemented among older adults living in a suburb of Stockholm. All interviews except the one on phone were conducted at the participating primary health care center within one month after finishing the intervention. To be able to catch the complexity of participation in an intervention including technology for persons at older ages, questions on aging in general and how old age affects daily activities were included in the interviews.

**Table 1:** Characteristics of the nine participants

Participant ID	Gender	Age
1	Male	74
2	Male	78
3	Male	74
4	Male	77
5	Male	76
6	Female	84
7	Female	75
8	Male	85
9	Male	85

### Data collection

The nine semi-structured interviews were conducted in Swedish (8 by LT and 1 by MT). The research team developed the interview guide (see appendix 1), which consisted of questions about the participants’ experiences participating in the prevention program and the use of technological tools. Observations and reflections captured during the interview were written directly after the interviews. The recorded interviews lasted between 31 and 40 minutes and were transcribed verbatim in the order they were conducted. ID numbers were assigned to each participant to maintain privacy and confidentiality. In addition, one interview was conducted with a nurse who had been active in delivering the intervention to strengthen the contextual understanding of the project for the authors of this paper, but this interview was not included in the analyses. Despite the limited number of interviews and relatively short duration, the data were considered to have enough information power for a rigorous analysis [32].

The reporting process followed the COnsolidated criteria for REeporting Qualitative research (COREQ) guidelines [33].

### Analysis

Two of the researchers (LT and MT) coded the interviews independently using the principles of qualitative content analysis [28, 29]. Both researchers had previous experience of conducting qualitative content analysis in a similar manner. Initially, five of the transcripts were coded in the qualitative software program NVivo 10, while open coding by hand was used for the rest of the interviews by both researchers. The coding was conducted on a manifest level to avoid interpretation at this stage. The researchers met for discussions where they agreed on how meaning units had been condensed into codes. The wording of the codes was revised when agreeing for a need clarification. This was for strengthening the common understanding and to reduce the number of codes before categorization. The categorization was done together by LT and MT. After the coding and categorization, all three researchers discussed the findings, and finally, two common themes with five categories were developed.

## Results

The analysis of the text resulted in two themes with two and three categories.

The two identified themes were as follows:1) Perceptions of being old are seldom positive, and 2) A rewarding/fruitful participation in the project with suggestions for improvement.

**Theme 1***-Perceptions of being old are seldom positive* was developed from the following categories: (1) Diminished health (physical and mental health) due to aging; and (2) Loss of former activities leading to passivity and feelings of reduction in quality of life.

This theme reflects how an older body becomes frailer and more exposed to pain than is experienced at younger ages.

### Diminished physical and mental health due to aging

Both physical and mental health were talked about as having decreased markedly at older ages. Many participants expressed physical shortcomings and other issues, such as tiredness and worries about sickness due to aging, showing a rather pessimistic view of becoming older. Previous routines of being physically active were expressed as being changed and replaced by less active activities and engaging in former activities of passion was no longer possible. Passivity at times described as related to an operation or other health-related issues, such as pain and feelings of sadness. The following quote from a 74-year-old man illustrates how activity levels can change due to aging:

… *before, I ran about three miles a week. In addition, today I do almost nothing, I walk. I cannot run either because I have full prostheses in each knee. (…) I want to run, that is what I like best. But now I’m walking instead. But yeah, it feels like it’s not the same…Well, there are endorphins that are released when you run.* (ID: 1)

In addition, many of the participants were worried and mentally stressed about not having many years left to live. There were several examples where cancer was a common reason for losing a close friend and cancer was talked about as a fear that could hit you or your close ones anytime. This loss served as a reminder of the older age they were in:

…*the only thing you want in the ages of seventy and above, there are many who died in my networks, from cancer and things, truly close friends. It worries me. I’m maybe, in some way preparing myself, if something happens suddenly, how would I take it, personally or in relation to my wife, It’s reality and facts, that something will happen, you cannot live forever, but you have to be prepared for these things, I think about them, most of the time.* (ID: 2)

Although few, some examples could be seen in the relief of getting older. This was often connected to not working any longer. Worklife was expressed as stressful compared with retirement, as illustrated in the following quote by a 74-year-old man:

*Well… I think it is quite nice (laughs). Sometimes you have worked and so for fifty years… Had worked at the same place for fifty years on that day yes, so then after fifty years; quit and went home… But it is like that, you sat like… ah, like a spider in a net at work… the phone was ringing all the time.* (ID: 3)

More negative experiences of being older regarding health were apparent. Although well-being in terms of not being stressed about work was positive, there were other examples of losing the social network that the work was offering, which was less positive.

### Loss of former activities leading to passivity and feelings of reduction in quality of life

The number of activities that were planned per day was markedly fewer than before. It was also expressed that getting started with activities took much longer than earlier, and it was common to plan for a longer period and to postpone the start of activities such as cleaning at home. The main reason for this was a lack of energy and strength that would have been required to manage the schedule as it would have been planned for earlier in life. Most of the participants described this infirmity as a hindrance to conducting daily activities. One 77-year-old man explained the situation in the following way:

*It’s that you cannot do so much in a day, you cannot handle so much. It’s what I assume… for daily life when you… can do two or three activities and you don’t have strength to do anymore.* (ID: 4)

Although the same participant quoted above talks about a friend of the same age who still can manage a full-time job:

*Ah, it is an awfully big difference. But there is also a person at same age as me who, who still works full-time, right… so there are differences, I would not be able to do so.* (ID: 4)

Another reason often described as hindering engagement in activities was pain. There could also be stress about getting hurt if they engaged in former physical activities because the body was considered frail compared to before, and they were less likely to take risks. This led to former meaningful activities no longer being joyful as explained by a 75-year-old woman:

*…I was in the garden a lot last spring. But then I haven’t had the strength to do it, but it could also be this ache that I have had that… you don’t feel for it. It takes, you lose the desire… when you don’t… when it hurts. I think it affects me a lot.* (ID: 7)

In addition, passivity due to aging was described, where aging itself was seen as a reason for sitting still more and not moving around as earlier in life. As illustrated in the following quote by a 78-year-old man:

*Well, I can say that, you have become more like… I don’t know how to say. You probably stay at home more and sit inside when you get older… you don’t go out and move around as much then… it must be the age (laughter).* (ID: 9)

In general, aging was associated with negative perceptions due to tiredness and pain leading to passivity and a feeling of lower quality of life than earlier in life.

**Theme 2 -***A rewarding and fruitful participation in the project with suggestions for improvement* was developed from the following categories: (1) Positive experiences of participation in the project; (2) Nonfunctioning components of the project with suggestions for improvement; and (3) Consequences of the participation (both positive and negative).

### Positive experiences of participation in the project

The participants were overall positive to have enrolled in the prevention program. One element often mentioned was the interaction with the research team and health professionals from the primary health care center involved in the project. The availability of health care staff and the researchers was highly appreciated:

*Yes, I could pick and choose, so it was yes… I had the technician all the time to help if I missed something so yes, I just called him to ask. So he has been very helpful.* (ID: 6)

Overall, the participants expressed positive experiences participating in a structured program. This is both in terms of physical and mental satisfaction. The involvement could at times feel like a reminder of how it was back at younger ages being involved in organized activities, as explained by a 78-year-old man:

*Well, I feel satisfied. When you get something done, combined with physical activity, then it’s great. I trained a lot in my youth, up to maybe forty years old, right, yes, I was active all the time with training and everything like that, so that, uh, not the same level, but roughly in comparison with this, I did something that I had done before to follow a program. Mentally, it was satisfying for me as well.* (ID: 2)

In general, it was reported that it had been a positive experience to be introduced to technical tools such as learning how to use a tablet. However, some obstacles could be noted in the process of learning how to use it. But there were also examples of having already integrated a tablet into a daily routine of reading the newspaper from a screen instead of in paper format.

### Non-functioning components of the project with suggestions for improvement

Although the interactions with the research team and the collaboration of health care personnel from primary care were highly acknowledged and appreciated, some parts did not work as expected. Sometimes the exercises were at the wrong level, and some critique of the program was expressed. Some of the exercises seemed to be too easy for some of the participants, who wished to be more challenged physically:

*…the exercises are too simple, some of them. Like walking in figure eights. But I understand that if you get older it’s hard to keep your balance sometimes (…) However, I think some exercises are too easy, but instead of twenty times, I have probably done them thirty, forty times and these liftings I have also done extra. They’re only five times a week, so you have two days for rest, and I have done them too.* (ID:1)

The suggested exercises from the Vivifrail program were described as too time-consuming for some of the participants, requiring more time than it was expected to take. In the following quote, one man explains how the exercises take more or less a whole day to get through:

*Most of all, it’s a too big part of the day, right? I do not know what I should have resigned or to rationalized it. I have not exactly been careless. But… been very… slow… I have taken… breaks between, between these steps. Often used to do that yes… took three exercises at a time so to speak… yes maybe three exercises… in the morning and three… or four…in the afternoon. Yes, but all in all, it was about that estimate. (…) It is simply too extensive.* (ID: 8)

The technical tools used for the measurements were often talked about as being of bad quality despite the help opportunities from the members of the project. This was because the tools did not always work as they were supposed to, which led to some of them not trusting the results from the measurements. Some measurements had to be done many times in a row to obtain the results that needed to be reported through the tablet, which could at times be frustrating. It was also confusing when, for example, the weighing scale provided by the project was not synchronized with one’s own weighing scale as described by a 77-year-old man:

*…I think it weighed a little differently each time. I have my own scale too, so they didn’t weigh… in the same way (…) then it was… this kneeling thing here, it was pretty good, I thought you were supposed to rise up, although sometimes, it measured wrong number of times, so…* (ID:4).

The level of using technical equipment such as computers before enrolling in the program differed between the participants to a large extent. Some of them used computers daily to search for information and send emails, and they were used to book health-related appointments online, while others had very little experience with digital communication as the POSITIVE system provided.

Many of the participants had no previous experience using a tablet, and although most of them acknowledged it was good to have the opportunity to familiarize themselves with the new technique, they sometimes had problems with using it. An 84-year-old woman who was not familiar with tablets from before, explains the issues with learning how to use them as follows, with a wish for a better introduction to how to use it:

*It went well, but yes, I think the letters are not all there, where I’m used to having them. Therefore, at the beginning, I wrote wrong, and then I did not know how to remove that letter that was wrong. it takes a little time to find exactly the letter I want, it was a bit slow because… when I wanted to have a full stop or comma to come… I could not find it. However, then my fingers were fast, so then I mistyped, I could not delete what I had written. No, yes, yes I think I received a bit of… bad information here, about how it was.* (ID: 6)

While some of them did not have any problems with the use of the tablets, they were still slightly critical of how the computer program in the tablet was organized, wishing for more simplicity and requiring fewer answers:

*I think it’s a bit… clumsy, and then also the one you can press on that tablet, they’re a lot so completely unnecessary there. Back and forth back and forth. About the same thing every time, no, I think it’s a bit clumsy done, you could have done much simpler, better I think. It is clumsily designed. (…) There has been a lot of unnecessary clicking and so…* (ID: 4).

In addition to the use of a tablet, other equipment also seemed to be slightly too complicated for some of the participants, leading to the postponement of the exercises:

*From start, I did it on Monday, from beginning, all those exercises in that one (the program), but I thought it was a bit of a hassle to deal with those cords and stuff, so I put it off until the weekend…* (ID: 4).

Sometimes straightforward suggestions for improvement of the program were shared. This 75-year-old woman would have wanted clearer explanations of why they should perform the exercises instead of only giving instructions on how:

*I do not know if it’s that, that when… when they have written these descriptions of… in book, the booklets. How you make these… moves. Then I think it’s great if they can write why you should do them. Because it is not there. It only describes.* (ID: 7).

A wish to meet other people in the same situation was also expressed, pointing to a curiosity about meeting other participants involved in the project:

*I can think that after three months, you could have met this group sometime. Who is joining? Yes, ask questions, what do you do? … which I don’t do (…) …if you look a little, what kind of people have joined, how many kilos overweight are they or how vigorous are they, or what kind of illnesses do they have?* (ID:1)

In general, the positive elements of adding technical equipment to participants’ daily lives seemed to be overshadowed by the problems experienced when using the devices. The social aspect of having contact with other participants was lacking and was experienced as something that would have added value to the program if implemented.

**Consequences of the participation (both positive and negative).**

Most participants said that participation in the program motivated them to start exercising and led to more physical activity in their daily lives. Increased feelings of strength in the legs and stomach were reported. It was also common that participants had increased their levels of outdoor walking, and some of them recorded their steps. Some other experienced outcomes are illustrated in the following quote:

*Yes, it’s a big difference, so it is… that I feel more agile. I’m more mobile, agile and so on… However, But yeah… I don’t think the balance has improved that much*. (ID: 4)

Increasing physical activity in daily life was experienced as positive and having the opportunity to perform the exercises in the participants’ home environment was highly appreciated. The participant below also expressed that it can be easier to continue the exercises when they can be performed from home:

*I think it has been positive, I must say because… then I have sort of started with… these kinds of… went, gym or exercise overall, at home, because before I have been at the gym. In addition, it has always, always ended with you quitting, after a while. However, at home then… there is a chance that you can continue right… I think it is great.* (ID: 4)

One 78-year-old man who initially considered the level of the exercises provided by the program to be too low level had modified the exercises to be more physically demanding. This resulted in the participant being injured from participation in the program. He explains it as follows:

…*the one to twist and lift, what’s it called… water bottles. Instead, I took dumbbells, I thought it was a bit… silly to have the two. I had dumbbells at home. But, maybe they were a little heavier, because it didn’t say how much it would weigh and so on, I was used to working with them before, so I took my old weights, maybe they were too much. Oh then, twisting the towel, it caused me some kind of inflammation, external, er, hand…, arm fold here. And it stopped me for a while but I continued by kind of not straining so much.* (ID: 2)

The same participant wanted to receive care through the project due to pain caused by the exercises and was frustrated about the care process through an ordinary primary health care center:

*I tried to get help, wrote to the nurse and they have advised me to sort of seek help from the health care center, but, then it was very difficult to get appointments with orthopedists and stuff like that. Oh well, at least I got an appointment for a shot (vaccination), but the follow-up never came because, they wanted me to wait until September or something; they did not have time. I think that they are a bit, er, you should take care of, in a different way, if something happens, during these trainings, or the exercises. That you would get a little, faster help.* (ID: 2)

The participants believed that the program could contribute to increasing the level of physical activity in their daily lives. At times, the exercises were not experienced to be on the right level. The possibility of adjusting the exercises was experienced as good but could potentially have harmful consequences.

## Discussion

The focus of this study was to describe the experiences of prefrail and frail older persons participating in a program in which the POSITIVE system was used to increase physical activity in their daily lives. The main findings were that, despite overall positive responses to participation in the program, there was still room for improvement, especially for the technical tools, which were perceived as being of low quality and at times difficult to use. Aging in general was perceived as linked to worse health with tiredness and pain and was associated with lowering the quality of life.

The first theme highlighted perceptions of becoming older as seldom positive included a loss of meaningful activities connected to increased feelings of loneliness. The interactions with the research team and the collaborating healthcare personnel from primary care were highly acknowledged and appreciated. Participation in the program added purpose to their everyday lives, adding a sense of coherence and belonging to the context. Taking care of one’s health was expressed as meaningful. In addition, the possibility of meeting other participants was requested. The appreciation of interactions with the project team and requests to meet other participants can be related to loneliness and a need to broaden the social network. There is extensive evidence of peer support for self-management [34] and of the value of social interaction for well-being [35, 36], which could be confirmed by our study results. Concerns were raised that it was difficult to book appointments at primary health care centers, which could be another reason why the relations with the people involved in the project were highly valued.

The other theme was revealing participation in the intervention as rewarding and fruitful, although there were room for improvement. The technical issues hindered the optimal use of the equipment provided for use in the participants’ home environment. The struggle to use the technical tools provided as part of the intervention is in line with previous research on the difficulties associated with the use of technical devices for the target group [18, 21, 22]. However, there is research showing that interventions that include physical activity in combination with the use of technology have the potential to prevent disability in older persons with frailty [37, 38]. The duality in both having struggles with the technical tools provided and seeing benefits in the use of them is confirmed by other qualitative studies [39]. Digital skills training focusing on the older persons’ lifeworld has been suggested [40] and this need and potential benefits can be confirmed by our study as well.

Participating in a physical activity intervention has been shown to have health benefits for frail older persons [5, 9, 11–13]. Although a systematic review by Clegg et al. (2012) [16] could not conclude that quality of life improved significantly, our results indicate that the intervention had a positive impact through engagement and attention received by participating in the program. Being able to engage in exercises from home was appreciated and talked about as improving the possibility of continuing to be physically active after finishing the intervention. This finding confirms that home-based exercises are applicable and feasible interventions for preventing a decrease in dependency in the study population [16].

The overall positive experience of participation indicates that the POSITIVE system might be an acceptable intervention for prefrail and frail older adults in Sweden. A more quantitative investigation is needed, as our qualitative study cannot be representative of older adults even in the Hässelby area. However, the development of technical solutions runs fast and future qualitative studies on participation in similar interventions including technical tools for older persons are therefore needed.

A strength of the study is that almost all participants who have finalized the program were included in this qualitative interview study where they had the chance to reflect on their participation in the intervention group. Another strength of using qualitative methods when evaluating randomized controlled trials such as the POSITIVE program is to get the participants’ perspectives for a deeper understanding of how the participation have been experienced. However, some limitations of the study should be highlighted. The positive responses could have been affected by selection bias since the participants who were invited to the program took the initiative to participate and showed interest in increasing physical activity in their daily lives to reverse and prevent further development of frailty. Many of the participants also expressed that they had experience and an interest in technical devices. This could be due to the study sample in which the majority were older men. For this older generation, men have worked in areas connected to technique to a larger extent than women in general in this age group. It could also be that this research project appeared more appealing to men due to the technical components. Participation in the intervention could also have been more appealing to participants due to the ongoing COVID-19 pandemic when other activities and social occasions were restricted. The results could also have been affected by the fact that the researchers interviewing the participants could have been associated with the project, leading to skewed positive responses in line with social desirability bias [41]. In addition, the researchers’ occupational backgrounds, two occupational therapists (LT and SG), and an academic coordinator (MT) could have influenced the study. In particular, the interest in activities and participation could have led to a specific framing with regard to this topic.

## Conclusion

Becoming older means a loss of meaningful activities and social relationships. Pain is common, and there is the stress of getting ill and losing loved ones. A physical activity intervention motivates activity and adds a sense of coherence by participation in the program increases social relations. Adding opportunities for participants to meet each other is suggested for improving the intervention in terms of increasing social dimensions. Our findings conclude that despite difficulties with handling the technical equipment, participation in the POSITIVE prevention program was in general a positive experience. A suggestion for improving the intervention is to add a social component for the participants to meet each other within the program.

### Electronic supplementary material

Below is the link to the electronic supplementary material.

Supplementary Material 1

## Data Availability

This study is built on qualitative interview data. All material is available on request from the corresponding author.

## Abbreviations

ADL: Activities of Daily Living
APHCC: Academic Primary Health Care Centre
COREQ: : COnsolidated criteria for REporting Qualitative research
