# Supplementary material for: POSITIVE: experiences of an intervention aiming for reversing and preventing frailty using a home monitoring and communication platform within primary health care
Source: BMC Geriatr. 2024 Apr 30;24:382. doi: 10.1186/s12877-024-04990-7 (PMC11061984; doi:10.1186/s12877-024-04990-7)
Supplement: Supplementary file 1 — Supplementary Material 1 [file 12877_2024_4990_MOESM1_ESM.docx]

**Intervjuguide med frågeområden för projektdeltagare - äldre i interventionsgruppen**

Du har deltagit i projektet POSITIVE där vi har använt teknik för att understödja hälsa och fysisk aktivitet för äldre och deras närstående som bor hemma under 6 månader. Vi skulle avslutningsvis vilja prata om dina erfarenheter av att delta i projektet och den här tekniken.

**Deltagande i ett forskningsprojekt**

- Skulle du kunna börja berätta lite övergripande om din upplevelse av att ha deltagit i projektet?
- Hur kom du i kontakt med projektet och sedan bestämde dig för att delta?
- (Kan du berätta om hur din närstående har deltagit och varit involverad?) *Till de som haft närstående*
- Finns det något som har varit en utmaning/svårt och finns det något som du upplever har fungerat bra och som kan ha underlättat för dig i din vardag?

**Upplevelse av åldrandet**

- Kan du berätta för mig om och hur du tänker kring att bli äldre och hur påverkar det ditt dagliga liv?
- Kan du berätta om det finns några speciella aktiviteter som har blivit påverkade av att du har blivit äldre?
- Hur har det varit att genomföra Vivifrail träningsprogram, upplever du någon förändring i din fysiska eller psykiska hälsa och i så fall kan du berätta hur?

**Studiedesign**

- Hur har du upplevt de olika testtillfällena under studien? (ex, var de långa, uttröttande, eller roliga..) (både teknikfrågor och fysiska tester..)
- Hur har du upplevt informationen du fått och kommunikationen med forskningsgruppen och den medicinska personalen under studien?

**Teknikanvändning**

- Kan du berätta om du använder någon form av vardagsteknik (ge exempel)? Exempelvis skickar du e-post, hanterar någon form av e-hälsa, mina vårdkontakter? Om du använder, använder du en dator och mobiltelefon i din vardag och i så fall till vad?
- Hur har du upplevt de tekniska lösningarna som du har använt i projektet?
- Kan du berätta om hur det har varit att hantera den tekniken som har varit involverad? -uppresningsmätaren, gånghastighetsmätaren, vågen och applikationen i surfplattan?
- Finns det någon situation som du kan beskriva när den här tekniken har underlättat för dig i din vardag? (exempelvis i kontakt med vården etc..)
- Kan du beskriva om eller hur teknikanvändningen har upplevts som hindrande för dig (och din närstående)? (exempelvis i kontakt med vården etc..)
- Hur skulle du vilja fortsätta att använda tekniska lösningar i din vardag?
- Finns det någon ytterligare information som du skulle vilja delge mig?
- Hur ser du på en fortsättning med systemet Positiv och dess innehåll som Vivifrail, hemtester och kommunikation med vårdpersonal?

STORT TACK!
